# Effects of Exercise on Dynamic Balance in People with Type 2 Diabetes Mellitus: A Systematic Review and Meta-Analysis of Randomized Controlled Trials

**DOI:** 10.3390/life15060913

**Published:** 2025-06-04

**Authors:** Wenda Zheng, Hao Su, Bing Han, Zhuying Chen, Xiaojie Liu, Yuanyuan Lv, Laikang Yu

**Affiliations:** 1 Beijing Key Laboratory of Sports Performance and Skill Assessment, Beijing Sport University, Beijing 100084, China; zwd669966@163.com; 2 Department of Strength and Conditioning Assessment and Monitoring, Beijing Sport University, Beijing 100084, China; 15662755237@163.com (H.S.); 13520753687@163.com (B.H.); zhuying20232120126@126.com (Z.C.); 3 Department of Pharmacology and Toxicology, Medical College of Wisconsin, Milwaukee, WI 53226, USA; xiaojieliu@mcw.edu; 4 China Institute of Sport and Health Science, Beijing Sport University, Beijing 100084, China

**Keywords:** exercise, dynamic balance, type 2 diabetes mellitus, multicomponent training

## Abstract

This study aimed to investigate the impact of exercise on the dynamic balance in type 2 diabetes mellitus (T2DM) patients and identify the optimal exercise prescription for clinical practice. A comprehensive search was conducted across the Web of Science, Scopus, Embase, Cochrane, and PubMed databases up to 10 November 2024, to identify randomized controlled trials assessing exercise interventions in T2DM patients, with dynamic balance as the primary outcome. Thirteen studies involving 413 participants were included. A pooled data analysis demonstrated that exercise significantly enhanced the dynamic balance (standardized mean difference, SMD, −0.50; *p* < 0.0001, I^2^ = 59%). The subgroup analyses revealed that multicomponent training (SMD, −0.84; *p* = 0.006, I^2^ = 76%), a frequency ≥ 3 times per week (SMD, −0.68; *p* < 0.0001, I^2^ = 57%), a session duration < 60 min (SMD, −0.52; *p* = 0.001, I^2^ = 67%), a weekly time ≥ 180 min (SMD, −0.64; *p* = 0.003, I^2^ = 65%), and supervised exercise (SMD, −0.59; *p* < 0.00001, I^2^ = 49%) were most effective. These findings suggest that supervised, multicomponent training performed at least three times weekly, with each session lasting <60 min, to attain a cumulative weekly time of 180 min, represents an evidence-based strategy to improve the dynamic balance in T2DM patients.

## 1. Introduction

The escalating prevalence of diabetes mellitus and its complications has emerged as a pressing global public health issue. According to the International Diabetes Federation, the number of individuals affected by diabetes has surpassed 536.6 million globally [1], with forecasts indicating a potential surge to 642 million by the year 2040 [2,3]. Type 2 diabetes mellitus (T2DM) is characterized by metabolic disturbances, primarily centered on impaired insulin secretion and heightened blood glucose levels [4], which can escalate the risk of severe complications. These complications encompass vision impairment, extremity amputations, diabetic peripheral neuropathy (DPN), chronic kidney disease, vasculopathy, and cardiovascular disorders [5]. Notably, diabetic retinopathy and neuropathy stand out as the most frequently occurring complications [6], frequently culminating in sensory and motor deficiencies. Such deficits are instrumental in precipitating mobility challenges, aberrant gait patterns, and balance disorders [7,8,9,10].

The ramifications of balance impairment in individuals afflicted with T2DM are especially disconcerting. Falls represent a primary etiology of disability and preventable fatalities among older adults. Epidemiological evidence reveals that within the diabetic population aged 65 and above, the incidence of falls reaches 39%, marking a threefold increase compared to their non-diabetic counterparts [11,12]. Dynamic balance, which can be conceptualized as the capacity to preserve postural stability during motion or in reaction to external forces, plays a pivotal role in fall prevention. A compromised dynamic balance stands as a critical risk factor for falls, underscoring the imperative for efficacious interventions to mitigate this concern [13].

Exercise has garnered widespread recognition as a potential modality to enhance balance and diminish fall risk. Previous studies have demonstrated that combined balance and resistance exercise can enhance physical balance and strength [14,15,16,17]. For example, Tai Chi has been shown to improve static balance and gait velocity in older adults [18,19,20]. Nevertheless, its efficacy specifically in diabetic populations remains underexplored. Similarly, sensory–motor training has shown limited impact on postural balance in T2DM patients without clinical signs of diabetic distal polyneuropathy [21]. Conversely, resistance exercise has demonstrated a moderate improvement in the balance and walking ability in elderly T2DM patients with sarcopenia [22]. Additionally, studies comparing ball exercises and Frenkel exercises suggest that the former may be more effective in improving balance in diabetic patients with peripheral neuropathy [23].

**Figure 1:**
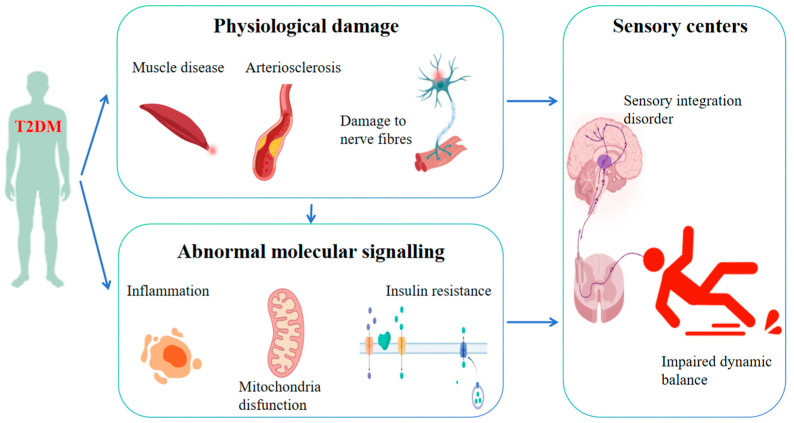
Mechanisms underlying the impact of T2DM on dynamic balance.

As depicted in Figure 1, T2DM instigates a systematic disruption of the dynamic homeostatic regulatory network via a tripartite cascade encompassing physiological structural damage (neurological, vascular, and muscular), dysfunctional sensory–central–motor circuits, and aberrations in molecular signaling (oxidative stress, inflammation, and insulin resistance). Research has elucidated that hyperglycemia inflicts damage upon the sensory nerve fibers in the lower limbs by activating the polyol pathway and amassing advanced glycosylation end-products (AGEs). This cascade of events results in diminished proprioception and vibratory sensation, thereby dismantling the sensory foundation essential for balance regulation. Consequently, patients encounter difficulties in precisely discerning the position of the foot and ground reaction forces [24]. In addition, hyperglycemia instigates endothelial dysfunction within small blood vessels, culminating in diminished cerebral blood flow to the cerebellum, vestibular system, and basal ganglia, inducing diabetic cerebral white matter lesions and blood–brain barrier damage. This vascular compromise induces diabetic cerebral white matter lesions and blood–brain barrier disruption, resulting in delayed balance regulation [25]. Finally, insulin resistance exerts a detrimental effect on muscle protein synthesis, leading to weakened lower limb strength, compromised standing balance and gait stability, and an elevated susceptibility to falls [26]. This theory framework lays the groundwork for exercise-based interventions aimed at improving the dynamic balance in T2DM patients by reversing damage through the activation of pathways such as AMP-activated protein kinase (AMPK) and brain-derived neurotrophic factor (BDNF) [27,28,29,30].

Systematic reviews have explored the benefits of various exercise modalities, such as yoga, proprioceptive training, and aerobic exercises on health-related fitness outcomes in T2DM patients [31]. While these interventions have shown promise in improving muscle strength, balance, and cardiorespiratory fitness, the quality of evidence remains low, and the heterogeneity of exercise protocols and outcome measures complicates the identification of optimal interventions. The existing research has predominantly focused on static balance or specific exercise capacities in T2DM patients, leaving a notable lacuna in our comprehension of exercise’s influence on dynamic balance within this specific population.

To address this gap, we conducted this study to evaluate the impact of exercise on the dynamic balance in T2DM patients and to identify the most effective exercise modalities for improving the dynamic balance.

## 2. Materials and Methods

### 2.1. Design

This investigation adhered to the Cochrane Handbook for Systematic Reviews of Interventions [32] and the Preferred Reporting Items for Systematic Reviews and Meta-Analysis (PRISMA, 2020) guidelines [33]. The protocol was registered on PROSPERO (CRD42024599019).

### 2.2. Search Strategy

An extensive search was executed across five electronic databases: Web of Science, Scopus, Embase, Cochrane, and PubMed, from inception to 10 November 2024. Search terms included keywords and MeSH terms related to “exercise”, “balance”, and “type 2 diabetes mellitus” (Table S1). Additionally, manual reference checks of relevant reviews were also performed to ensure all related studies were included.

### 2.3. Eligibility Criteria

Inclusion criteria were as follows: (1) randomized controlled trials (RCTs); (2) both intervention and control groups included; (3) participants had a T2DM diagnosis; and (4) dynamic balance was a primary outcome.

Exclusion criteria included (1) non-English articles; (2) conference abstracts; (3) review articles; (4) studies with irretrievable outcome data for mean and standard deviation (SD) calculation; and (5) studies where the control group underwent exercise interventions.

### 2.4. Data Extraction

Two authors (W.Z. and H.S.) independently extracted data, covering the studies’ characteristics (first author’s, publication year, sample size), intervention details (intervention type, duration, frequency, session duration, weekly time), participant characteristics (disease duration, age), and treatment effect values reflecting the changes in balance post-intervention. Discrepancies were resolved through discussion with a third author (L.Y.).

### 2.5. Methodological Quality Assessment

The Cochrane Risk of Bias (RoB) tool was used to assess methodological quality [34], evaluating domains such as selection bias, performance bias, detection bias, attrition bias, reporting bias, and other biases [35]. Studies were rated as having a “low”, “uncertain”, or “high” risk of bias in each domain. Discrepancies were resolved through discussion with a third author (L.Y.).

### 2.6. Statistical Analysis

Changes in dynamic balance outcomes were analyzed using mean and SD values, with standard error (SE) or 95% confidence interval (CI) converted to SD when needed [36]. A random-effects model was applied for data synthesis and 95% CI calculation. For high heterogeneity (I^2^ > 50%), meta-regression, subgroup, and sensitivity analyses were performed [37].

Subgroup analyses were based on intervention type, frequency, session duration, weekly time, and supervision status. Statistical analyses were performed using RevMan 5.4 for generating forest plots, and Stata 17 software for funnel plots, meta-regression, sensitivity analysis, and Egger’s test, with *p* < 0.05 considered statistically significant.

## 3. Results

### 3.1. Study Selection

**Figure 2:**
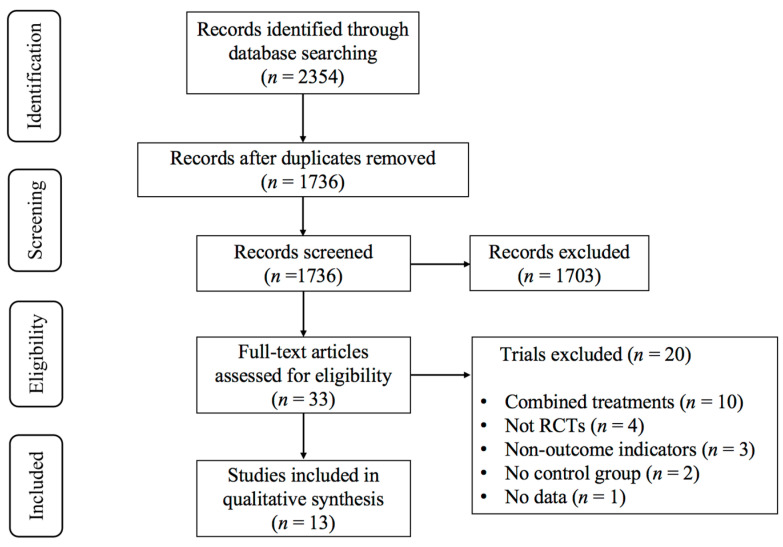
PRISMA flowchart of study selection.

An initial database search yielded 2354 articles. After removing duplicates, 1736 studies remained. The screening of the titles and abstracts resulted in 33 potentially eligible studies. Following a full-text review, 13 studies [23,38,39,40,41,42,43,44,45,46,47,48,49] examining the effect of exercise on the dynamic balance in T2DM patients were deemed eligible for inclusion. Figure 2 outlines the study selection procedure.

### 3.2. Characteristics of the Included Studies

The key characteristics of the included studies are summarized in Table S2. The 13 included studies comprised 413 participants in the intervention groups and 401 participants in the control groups. The exercise interventions varied, including aerobic, resistance, and multicomponent training, with durations spanning 3 weeks to 12 months. One study focused exclusively on women [42], fifteen studies included both men and women [23,39,40,41,43,44,45,46,47,48], and the remaining studies did not report the participants’ gender [38,49]. The mean age of the participants ranged from 52.82 to 74.29 years.

The dynamic balance was assessed using various outcome measures, including the Timed Up and Go Test (TUGT), Crossing Beam, Step Initiation and Gait (SIG), Walk Across, Tinetti Balance and Gait Test (TBGT), Functional Reach Test (FRT), 8-Foot Up and Go (8FUG), Star Excursion Balance Test (SEBT), Fullerton Advanced Balance (FAB), and Tandem Walk Score. Aerobic exercise was the most common intervention (11 studies) [38,39,40,45,46,47,48,49], followed by multicomponent training (5 studies) [23,41,42,44,46] and resistance exercise (2 studies) [23,46]. The intervention frequency ranged from two to five times weekly, averaging three times per week. The session durations varied from 40 to 120 min, with the weekly time ranging from 100 to 275 min.

### 3.3. Main Effect

**Figure 3:**
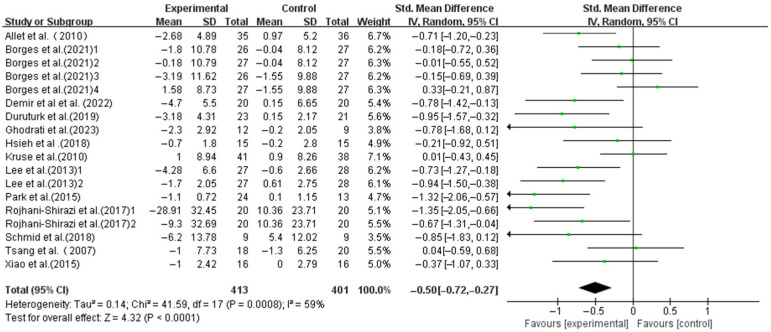
Meta-analysis results of the effect of exercise on dynamic balance in T2DM patients [23,38,39,40,41,42,43,44,45,46,47,48,49].

Exercise significantly improved the dynamic balance in T2DM patients (SMD, −0.50; 95%CI, −0.72 to −0.27; *p* < 0.0001, I^2^ = 59%, Figure 3). To investigate the heterogeneity sources, subgroup, meta-regression, and sensitivity analyses were conducted.

### 3.4. Subgroup Analysis

**Figure 4:**
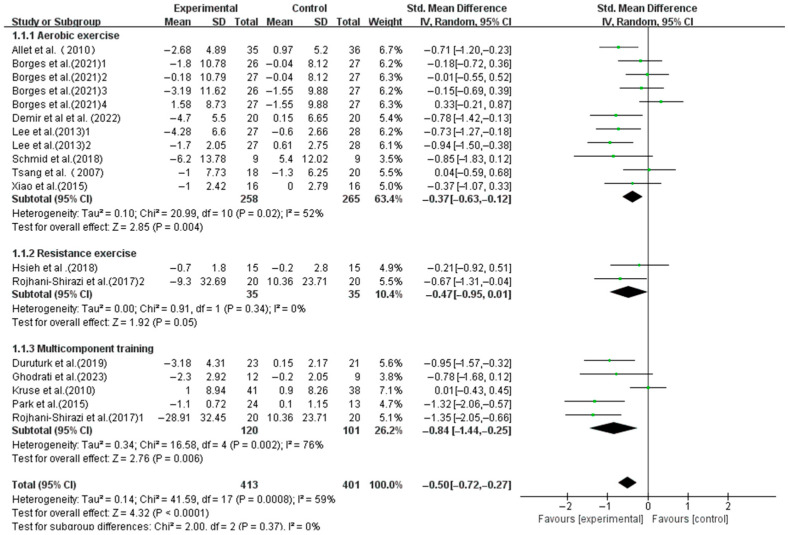
Meta-analysis results of the effect of types of intervention on dynamic balance in T2DM patients [23,38,39,40,41,42,43,44,45,46,47,48,49].

Aerobic exercise (SMD, −0.37; 95%CI, −0.63 to −0.12; *p* = 0.004, I^2^ = 52%) and multicomponent training (SMD, −0.84; 95%CI, −1.44 to −0.25; *p* = 0.006, I^2^ = 76%) significantly enhanced the dynamic balance in T2DM patients, with multicomponent training showing a greater effect. However, resistance exercise did not show a significant association (SMD, −0.47; 95%CI, −0.95 to 0.01; *p* = 0.05, I^2^ = 0%, Figure 4).

**Figure 5:**
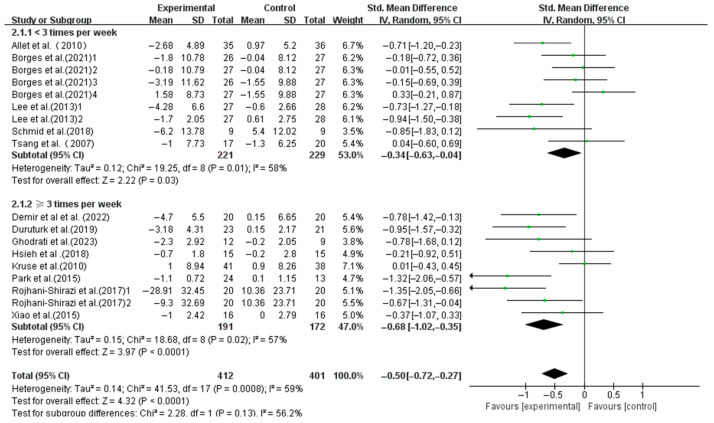
Meta-analysis results of the effect of frequency of intervention on dynamic balance in T2DM patients [23,38,39,40,41,42,43,44,45,46,47,48,49].

Interventions with frequencies < 3 times weekly (SMD, −0.34; 95%CI, −0.63 to −0.04; *p* = 0.03, I^2^ = 58%) and ≥3 times weekly (SMD, −0.68; 95%CI, −1.02 to −0.35; *p* < 0.0001, I^2^ = 57%) both significantly enhanced the dynamic balance, with higher frequencies yielding larger effects (Figure 5).

**Figure 6:**
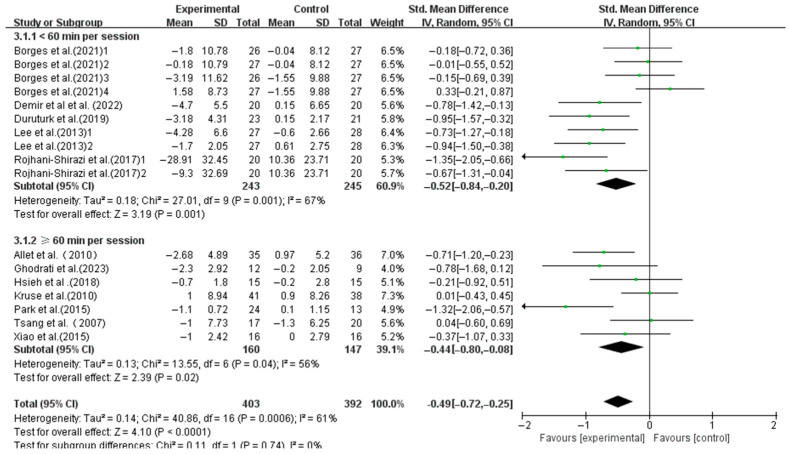
Meta-analysis results of the effect of duration of intervention per session on dynamic balance in T2DM patients [23,38,39,40,41,42,43,44,45,46,48,49].

Similar significant improvements were found in interventions with session durations < 60 min (SMD, −0.52; 95%CI, −0.84 to −0.20; *p* = 0.001, I^2^ = 67%) and ≥60 min (SMD, −0.44; 95%CI, −0.80 to −0.08; *p* = 0.02, I^2^ = 56%), with shorter sessions showing greater effects (Figure 6).

**Figure 7:**
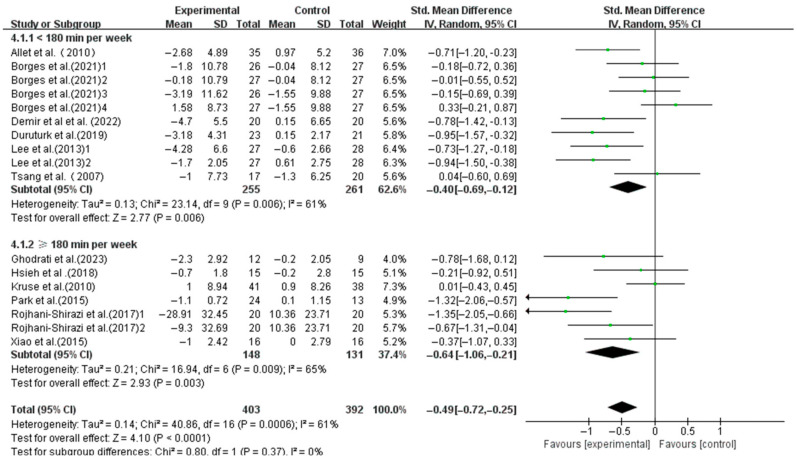
Meta-analysis results of the effect of duration of intervention per week on dynamic balance in T2DM patients [23,38,39,40,41,42,43,44,45,46,48,49].

The interventions with a weekly time < 180 min (SMD, −0.40; 95%CI, −0.69 to −0.12; *p* = 0.006, I^2^ = 61%) and ≥180 min (SMD, −0.64; 95%CI, −1.06 to −0.21; *p* = 0.003, I^2^ = 65%) both significantly enhanced the dynamic balance, with a longer weekly time showing greater effects (Figure 7).

**Figure 8:**
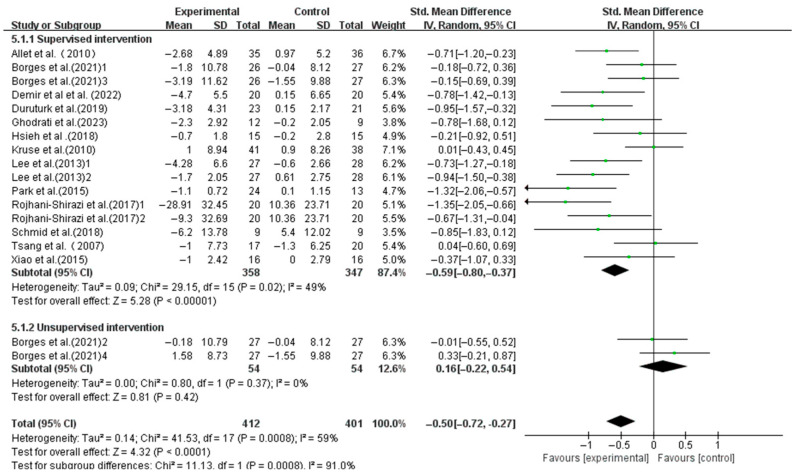
Meta-analysis results of the effect of supervised and unsupervised interventions on dynamic balance in T2DM patients [23,38,39,40,41,42,43,44,45,46,47,48,49].

Supervised interventions significantly enhanced the dynamic balance (SMD, −0.59; 95%CI, −0.80 to −0.37; *p* < 0.00001, I^2^ = 49%), whereas unsupervised interventions did not (SMD, 0.16; 95%CI, −0.22 to 0.54; *p* = 0.42, I^2^ = 0%, Figure 8).

### 3.5. Meta-Regression

The meta-regression analysis indicated that the frequency of interventions per week (*p* = 0.045), session duration (*p* = 0.019), and weekly time (*p* = 0.005) were significant moderators of the effect of exercise on the dynamic balance in T2DM patients (Table S3).

### 3.6. Risk of Bias

Using the RoB tool, the methodological quality assessment revealed seven studies with a low bias risk [23,40,41,43,44,49], four with a high bias risk [38,44,46,48], and seven with an unclear bias risk [39,45,46] (Figure S1).

### 3.7. Publication Bias

The funnel plot (Figure S2) and Egger’s test (*p* = 0.057, Table S4) suggested no significant publication bias, indicating that the results were not substantially influenced by small-study effects.

### 3.8. Sensitivity Analysis

The sensitivity analysis demonstrated the stability of the meta-analysis results. Excluding individual studies did not significantly alter the overall effect estimates, confirming the robustness of the findings (Figure S3).

## 4. Discussion

Our findings demonstrated that exercise significantly improved the dynamic balance in this population, with multicomponent training, a higher frequency (≥3 times per week), shorter session durations (<60 min per session), a longer weekly time (≥180 min per week), and supervised interventions yielding the most pronounced benefits.

### 4.1. Effects of Exercise on Dynamic Balance in T2DM Patients

This study revealed that exercise significantly enhances the dynamic balance in T2DM patients, aligning with prior research. For instance, Ahn et al. [50] reported that Tai Chi improves balance in diabetic patients with neuropathy, while Ng et al. [51] found that ankle-focused training enhances postural stability. Venkataraman et al. [52] demonstrated that structured strength and balance training reduces the fall risk in individuals with diabetic peripheral neuropathy (DPN). These findings underscore the potential of exercise to counteract the sensory–motor deficits associated with T2DM, which often lead to balance impairments and increased fall risk.

Maintaining balance involves complex interactions across multiple systems. Physiologically, balance relies on three key components: sensory input, central integration, and motor control. Human balance is mainly affected by sensory transmission pathways, and any disruption in these pathways can affect balance [53,54]. Sensory inputs include visual, proprioceptive, and vestibular signals. These sensory signals are processed and integrated by the spinal cord, vestibular nuclei, medial longitudinal fasciculus, brainstem reticular formation, cerebellum, and cerebral cortex. The resulting neural impulses transmitted via γ-motor fibers control the tension of intrafusal muscle fibers, while those via α-motor fibers regulate the contraction and relaxation of the skeletal muscles [55]. Additionally, balance is related to brain function. For instance, sustained Tai Chi training has been shown to augment the cortical thickness in the right precentral gyrus and enhance the homogeneity of the postcentral gyrus [56]. These neural adaptations may bolster the brain’s ability to coordinate muscles for better balance control [57]. Exercise also stimulates proprioceptors in the joint capsule, tendons, and muscles of the lower limbs, enhancing spatial awareness and improving lower limb balance [58,59,60,61]. Dixit et al. reported that exercise treatment increases nerve conduction velocity and reduces nerve deterioration, potentially explaining the physiological bias for improved balance outcomes [59].

However, Orr et al. [60] reported no significant differences in balance function between Tai Chi and control groups. This may be due to an insufficient training dosage (intensity, frequency, duration) or movement design in the “Tai Chi for Diabetes” program, which failed to trigger significant adaptive changes. Additionally, T2DM is a complex condition often accompanied by multiple complications such as obesity, cardiovascular disease, and neuropathy, which may interfere with the isolated effect of Tai Chi on balance function. Therefore, in developing exercise programs for T2DM patients, it is crucial to account for individual variability and the quality and consistency of the training regimen to optimize balance enhancement.

### 4.2. Effects of Different Exercise Modalities on Dynamic Balance in T2DM Patients

Our study provides evidence that exercise can improve balance in T2DM patients, but significant heterogeneity was observed. Subgroup analyses were conducted to explore the effects of the intervention types, frequency, session duration, weekly time, and supervised versus unsupervised training on dynamic balance.

Multicomponent training emerged as the most effective intervention strategy. This finding corroborates evidence from studies demonstrating that a combination of resistance and aerobic exercises can mitigate neuropathic symptoms, enhance muscle fiber density, and bolster walking strength and balance [61,62]. Morrison et al. [63] also reported significant improvements in walking, response time, balance indices, and dynamic position with combined resistance and aerobic exercise. These findings align with recommendations from the European Society of Cardiology [64], American College of Sports Medicine (ACSM) [65], Belgian Physical Therapy Association [66], and Exercise and Sports Science Australia [67].

Regarding frequency, interventions conducted three or more times per week significantly improved dynamic balance, while those conducted less frequently did not. This aligns with the guidelines from the American Heart Association (AHA) and ACSM, which advise that older adults participate in a minimum of 30 min of moderate-intensity exercise five days per week, alternatively, engage in at least 20 min of vigorous-intensity exercise three days per week [65,68]. However, frequency alone cannot fully account for the effects of exercise on dynamic balance, as other factors such as session duration may also influence outcomes.

In an expert consensus guideline on exercise for older adults, it was highlighted that exercise exhibits a dose–response relationship with health. Specifically, an appropriate exercise intensity and duration are essential for maximizing health benefits [69]. Conversely, excessively prolonged exercise sessions do not confer additional health advantages and may potentially exert negative effects on the body [70]. Our findings revealed that exercise sessions lasting less than 60 min were most effective in improving dynamic balance in T2DM patients, which is consistent with Abdelaal et al. [71]. In addition, Lesinski et al. [72] also found that exercise sessions of 31 to 45 min were most effective for overall balance performance, while durations exceeding 60 min provided no additional benefits. Brief exercise may not be sufficient to prompt alterations in physiological arousal, cerebral architecture, and cognitive function. Conversely, excessively lengthy exercise sessions can result in undue fatigue and may not catalyze structural adaptations within the organism [73]. Therefore, determining the most effective duration for such changes is crucial. Future research should explore the optimal exercise duration for inducing structural and functional changes in the body.

To offer novel perspectives for exercise prescription, we have computed the weekly time by factoring in both frequency and session duration. The World Health Organization (WHO) advocates for 150 to 300 min of moderate-intensity aerobic activity, 75 to 150 min of vigorous-intensity aerobic activity, or an equivalent mix per week [74]. Similarly, the ACSM posits that individuals with type 2 diabetes mellitus (T2DM) should accumulate a minimum of 150 to 300 min of moderate-intensity aerobic exercise weekly [68]. Our findings revealed that exercise interventions of at least 180 min per week were the most effective for improving dynamic balance in T2DM patients, consistent with Tan et al. [75], who reported positive effects on balance and related physical activities with 180 min of weekly exercise in older adults with T2DM.

Our subgroup analysis showed that supervised exercise significantly improved the dynamic balance in T2DM patients, while unsupervised exercise did not. Possible explanations for this situation include, on the one hand, that during the training process, supervision ensures that patients correctly execute the high-intensity training program and amplifies the training effect [76]. Supervisors can promptly correct patient’s training maneuvers and adjust the training intensity, thereby ensuring safe and effective training to better meet patients’ goals [77]. On the other hand, T2DM patients participating in supervised training demonstrate higher compliance and physical activity control compared to those engaging in unsupervised exercise training [65]. Therefore, supervised training is essential for optimizing exercise outcomes in T2DM patients.

### 4.3. Limitations

Several limitations should be noted. First, as the studies were RCTs, exercise interventions could not be blinded, potentially introducing bias during quality assessment. Second, many studies did not report exercise intensity, limiting our understanding of its effect on the dynamic balance in T2DM patients. Third, the included studies focused on T2DM patients without categorizing specific health complications, which may have influenced the results. Finally, significant heterogeneity in the meta-analysis results requires a cautious interpretation of our findings.

## 5. Conclusions

Exercise significantly enhances dynamic balance in T2DM patients, with multicomponent training emerging as the preeminent modality. This study furnishes healthcare providers with empirical support to advocate for supervised exercise regimens for T2DM patients, stipulating a minimum of three weekly sessions, each preferably less than 60 min in duration, with the goal of accumulating 180 min of exercise weekly. Future research should focus on exploring the optimal exercise intensity and duration to further enhance the dynamic balance in T2DM patients.

## Data Availability

All data generated or analyzed during this study are included in the article/Supplementary Materials.

## Supplementary Materials

The following supporting information can be downloaded at https://www.mdpi.com/article/10.3390/life15060913/s1: Figure S1: Results of Cochrane Risk of Bias tool; Figure S2: Funnel plot; Figure S3: Sensitivity analysis results; Table S1: Search strategies; Table S2: Characteristics of the studies included in this meta-analysis; Table S3: Results of meta-regression; Table S4: Results of Egger’s test.
