# Supplementary material for: Effects of Exercise on Dynamic Balance in People with Type 2 Diabetes Mellitus: A Systematic Review and Meta-Analysis of Randomized Controlled Trials
Source: Life (Basel). 2025 Jun 4;15(6):913. doi: 10.3390/life15060913 (PMC12194284; doi:10.3390/life15060913)

Supplemental material

Effects of exercise on dynamic balance in people with type 2 diabetes mellitus: a systematic review and meta-analysis of randomized controlled trials

Table S1 Search strategies 2

Table S2 Characteristics of the studies included in this meta-analysis 3

Table S3 Results of meta-regression 6

Table S4 Results of Egger's test 7

Figure S1 Results of Cochrane risk of bias tool 8

Figure S2 Funnel plot 9

Figure S3 Sensitivity analysis results 10

Table S1 Search strategies

**Exercise search terms combined with "OR"**
Physical exercise programs; Physical Therapy Modalities; Physical Therapy Modalities; Modalities, Physical Therapy; Modality, Physical Therapy; Physical Therapy Modality; Physiotherapy; Physical Therapy Techniques; Physical Therapy Technique; Techniques, Physical Therapy; Exercise Movement Techniques; Exercise Movement Techniques; Movement Techniques, Exercise; Exercise Therapy; Exercise Therapy; Therapy, Exercise; Exercise Therapies; Therapies, Exercise; Exercise, Physical; Exercises, Physical; Physical Exercise; Physical Exercises; Exercise, Isometric; Exercises, Isometric; Isometric Exercises; Isometric Exercise; Exercise, Aerobic; Aerobic Exercises; Aerobic Exercise; Resistance Training; Resistance Training; Training, Resistance; Strength Training; Training, Strength; Weight-Lifting Strengthening Program; Strengthening Program, Weight-Lifting; Strengthening Programs, Weight-Lifting; Weight Lifting Strengthening Program; Weight-Lifting Strengthening Programs; Weight-Lifting Exercise Program; Exercise Program, Weight-Lifting; Exercise Programs, Weight-Lifting; Weight Lifting Exercise Program; Weight-Lifting Exercise Programs; Weight-Bearing Strengthening Program; Strengthening Program, Weight-Bearing; Strengthening Programs, Weight-Bearing; Weight Bearing Strengthening Program; Weight-Bearing Strengthening Programs; Weight-Bearing Exercise Program; Exercise Program, Weight-Bearing; Exercise Programs, Weight-Bearing; Weight Bearing Exercise Program; Weight-Bearing Exercise Programs
**Balance search terms combined with "OR"**
Balance; Postural Balance; Posture Equilibrium; Equilibrium, Posture; Posture Equilibriums; Balance, Postural; Postural Equilibrium; Equilibrium, Postural; Posture Balance; Balance, Posture; Posture Balances; Musculoskeletal Equilibrium; Equilibrium, Musculoskeletal; Postural Control; Control, Postural; Postural Controls; Posture Control; Control, Posture; Posture Controls
**Type 2 diabetes mellitus search terms combined with "OR"**
Diabetes Mellitus, Type 2; Diabetes Mellitus, Noninsulin-Dependent; Diabetes Mellitus, Ketosis-Resistant; Diabetes Mellitus, Ketosis Resistant; Ketosis-Resistant Diabetes Mellitus; Diabetes Mellitus, Non Insulin Dependent; Diabetes Mellitus, Non-Insulin-Dependent; Non-Insulin-Dependent Diabetes Mellitus; Diabetes Mellitus, Stable; Stable Diabetes Mellitus; Diabetes Mellitus, Type II; NIDDM; Diabetes Mellitus, Noninsulin Dependent; Diabetes Mellitus, Maturity-Onset; Diabetes Mellitus, Maturity Onset; Maturity-Onset Diabetes Mellitus; Maturity Onset Diabetes Mellitus; MODY; Diabetes Mellitus, Slow-Onset; Diabetes Mellitus, Slow Onset; Slow-Onset Diabetes Mellitus; Type 2 Diabetes Mellitus; Noninsulin-Dependent Diabetes Mellitus; Noninsulin Dependent Diabetes Mellitus; Maturity-Onset Diabetes; Diabetes, Maturity-Onset; Maturity Onset Diabetes; Type 2 Diabetes; Diabetes, Type 2; Diabetes Mellitus, Adult-Onset; Adult-Onset Diabetes Mellitus; Diabetes Mellitus, Adult Onset

Table S2 Characteristics of the studies included in this meta-analysis

Study	Sample size	Age(y)	Intervention	Supervision situation	Session duration (min)	Frequency (times/ week)	Intervention duration (week)	Balance indicators
Allet et al.（2010）	INT: 35<br>CON:36	63±7.99<br>64±8.89	Aerobic exercise	Supervised intervention	60	2	12	Crossing beam
Borges et al. (2021)1	INT: 26<br>CON:27	56.8±6.1<br>55.7±5.4	Aerobic exercise	Supervised intervention	45	2	12	SIG
Borges et al. (2021)2	INT: 27<br>CON:27	55.8±6.2<br>55.7±5.4	Aerobic exercise	Unsupervised intervention	45	2	12	SIG
Borges et al. (2021)3	INT: 26<br>CON:27	56.8±6.1<br>55.7±5.4	Aerobic exercise	Supervised intervention	45	2	12	Walk across
Borges et al. (2021)4	INT: 27<br>CON:27	55.8±6.2<br>55.7±5.4	Aerobic exercise	Unsupervised intervention	45	2	12	Walk across
Demir et al et al. (2022)	INT: 20<br>CON:20	73.50±7.08<br>72.45±7.25	Aerobic exercise	Supervised intervention	45	3	8	TBGT

Duruturk et al. (2019)	INT: 23<br>CON:21	52.82 ± 11.86<br>53.04 ± 10.45	Multicomponent training	Supervised intervention	40	3	6	TUGT
Ghodrati et al. (2023)	INT: 12<br>CON: 9	58.8 ± 1.5<br>58.8 ± 1.5	Multicomponent training	Supervised intervention	65	3	12	TUGT
Hsieh et al. (2018)	INT: 15<br>CON:15	70.6 ± 4.2<br>71.8 ± 4.5	Resistance exercise	Supervised intervention	60	3	12	TUGT
Kruse et al. (2010)	INT: 41<br>CON:38	66.3 ± 10.6<br>64.8 ± 9.4	Multicomponent training	Supervised intervention	60	3	48	TUGT
Lee et al. (2013)1	INT: 27<br>CON:28	73.78 ± 4.77<br>74.29 ± 5.20	Aerobic exercise	Supervised intervention	50	2	10	FRT
Lee et al. (2013)2	INT: 27<br>CON:28	73.78 ± 4.77<br>74.29 ± 5.20	Aerobic exercise	Supervised intervention	50	2	10	TUGT
Park et al. (2015)	INT: 24<br>CON:13	71.2 ± 3.9<br>69.6 ± 3.6	Multicomponent training	Supervised intervention	60	3	12	8FUG
Rojhani-Shirazi et al. (2017)1	INT: 20<br>CON:20	55 ± 5.76<br>53.85 ± 5.57	Multicomponent training	Supervised intervention	55	5	3	SEBT

Rojhani-Shirazi et al. (2017)2	INT: 20<br>CON:20	54.15±4.52<br>53.85±5.57	Resistance exercise	Supervised intervention	55	5	3	SEBT
Schmid et al. (2018)	INT: 9<br>CON: 9	55.6±9.62<br>53.3±11	Aerobic exercise	Supervised intervention	NR	2	8	FAB
Tsang et al.（2007)	INT: 18<br>CON:20	66±8<br>65±8	Aerobic exercise	Supervised intervention	60	2	16	Tandem walk score
Xiao et al. (2015)	INT: 16<br>CON:35	65.5<br>65.5	Aerobic exercise	Supervised intervention	60-120	3	12	TUGT

Abbreviations: INT, intervention group; CON, control group; SIG, stability in gait; TUGT, timed up-and-go test; TBGT, tinetti balance and gait test; FRT, functional reach test; 8FUG, 8 foot up and go; SEBT, star excursion balance test; FAB, fullerton advanced balance; NR, no report

Table S3 Results of meta-regression

Modalities	_ES	Coef.	Std. Err	t	p > |t|	95% CI
**Frequency**	subgroup	-0.3529965	0.226635	-1.54	0.144	-0.8398009, 0.1338079
	_cons	-0.3427468	0.1574143	-2.18	0.045	-0.6764503, -0.0090434
**Session duration**	subgroup	-0.235952	0.2538695	-0.93	0.367	-0.7770621, 0.3051581
	_cons	-0.4090075	0.15573	-2.63	0.019	-0.7409381, -0.0770769
**Weekly time**	subgroup	0.074195	0.2557145	0.29	0.776	-0.4788475, 0.6192375
	_cons	-0.527726	0.160205	-3.29	0.005	-0.8691948, -0.1862571

Abbreviations: Coef., coefficient; Std. Err., standard error; t, t-test statistic; p, probability; 95% CI, 95% cofidence interval.

Table S4 Results of Egger's test

Std_EFF	Coef.	Std. Err.	t	p > |t|	95% CI
Slope	0.6779112	0.5641463	1.20	0.247	-0.5180255, 1.873848
Bias	-3.783355	1.840496	-2.06	0.057	-7.685032, 0.1183215

Abbreviations: Coef., coeficient; Std. Err., standard error; t, t-test statistic; p, probability; 95% CI, 95% cofidence interval.

Figure S1 Results of Cochrane risk of bias tool

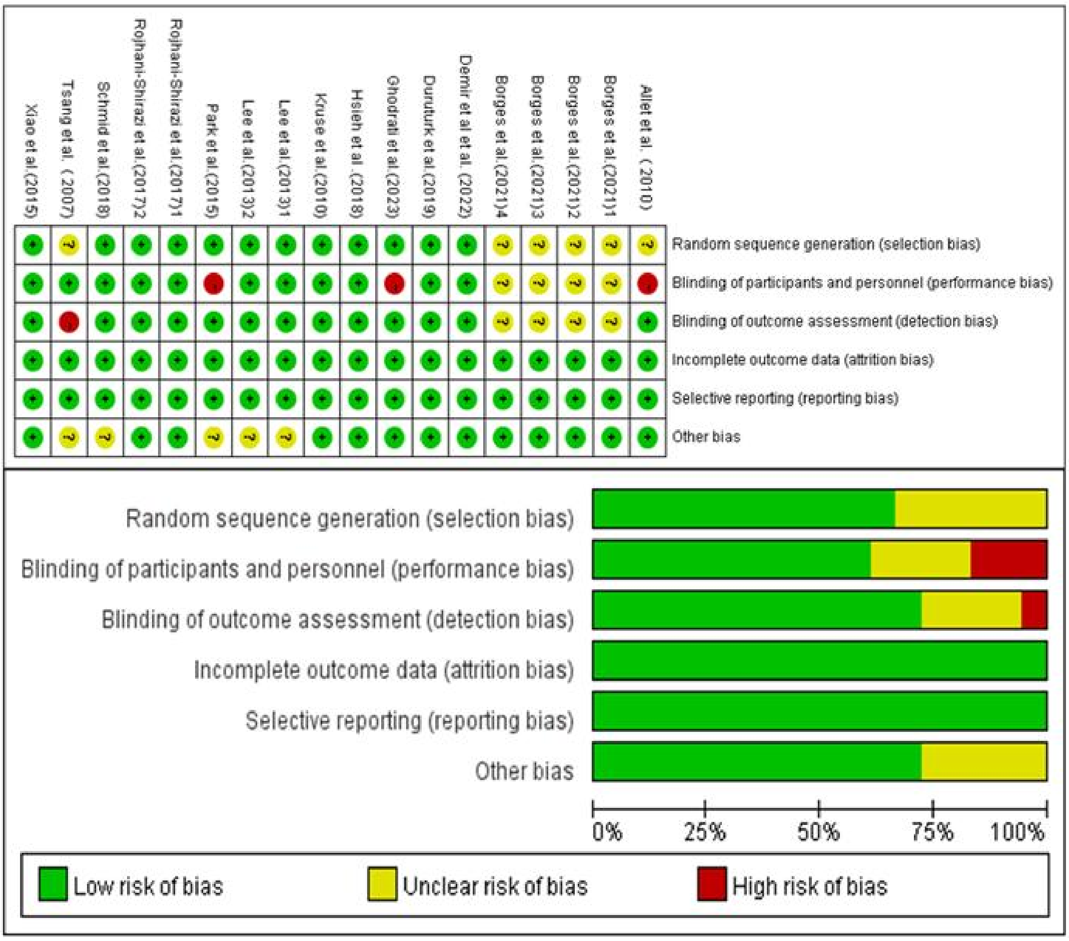

Figure S2 Funnel plot

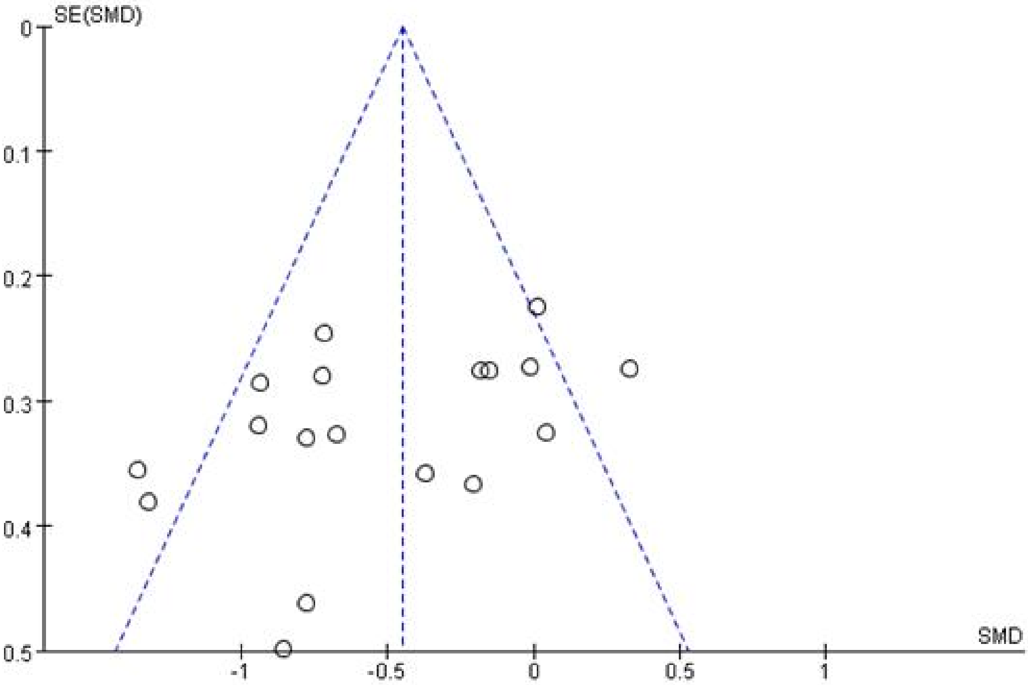

Figure S3 Sensitivity analysis

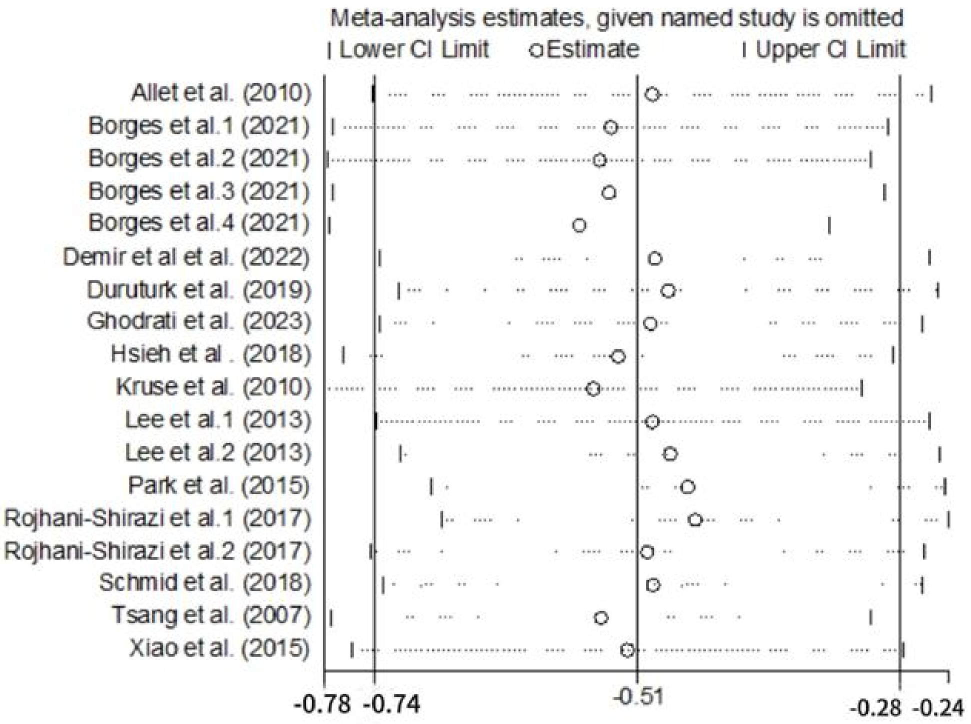

Supplement: Supplementary file 1 [file life-15-00913-s001.zip › life-3651001-supplementary.pdf]
